# The impact of visual and hearing impairments on the risk of arthritis among middle-aged and older Chinese adults (2011–2015): the mediating role of depressive symptoms

**DOI:** 10.1186/s12889-025-25495-x

**Published:** 2025-11-29

**Authors:** Chenyang Li, Shuai Ma, Ning Zhang, Jie Yuan, Tengbo Shi, Shanlin Tong, Ying Zhang

**Affiliations:** 1 Luoyang Orthopedic-Traumatological Hospital of Henan Province (Henan Provincial Orthopedic Hospital), Luoyang, China; 2 Fujian University of Traditional Chinese Medicine, Fuzhou, China; 3 Henan University of Traditional Chinese Medicine, Zhengzhou, China; 4 Zhengzhou Health College, Zhengzhou, China

**Keywords:** Sensory impairment, Visual impairment, Hearing impairment, Arthritis, Depressive symptoms, Mediation analysis, Aging population, CHARLS

## Abstract

**Background:**

To date, the mechanisms underlying the relationship between sensory impairment and new-onset arthritis remain inadequately understood. This study aims to investigate the mediating role of depressive symptoms in the longitudinal association between sensory impairment and new-onset arthritis, specifically focusing on visual impairment (VI), hearing impairment (HI), and dual sensory impairment (DSI).

**Objectives:**

This study aimed to elucidate the associative dynamics between various sensory impairments and the risk of developing new-onset arthritis, with a specific focus on the mediating influence of depressive symptoms. By understanding these interconnections, we seek to provide insights that could inform targeted interventions aimed at mitigating arthritis onset in older adults.

**Methods:**

Utilizing data derived from the 2011 China Health and Retirement Longitudinal Study (CHARLS), a cohort of 8,415 participants aged 45 years and older was analyzed. Participants were categorized based on their sensory status into four groups: no impairment, VI only, HI only, and DSI. A multivariate logistic regression analysis was employed to assess the association between sensory impairments and the incidence of arthritis over a four-year follow-up period, supplemented by subgroup analyses and mediation analysis to explore the mediating role of depressive symptoms.

**Results:**

Among 8,415 participants who were free of arthritis at baseline (2011), 1,554 developed arthritis by 2015. In fully adjusted models, visual impairment (VI), hearing impairment (HI), and dual sensory impairment (DSI) were each found to be associated with higher odds of incident arthritis (VI: aOR = 1.32; HI: aOR = 1.39; DSI: aOR = 1.34). In a four-category analysis, using individuals with no sensory impairment as the reference group, the results indicated that VI only (aOR = 1.29, 95% CI 1.07–1.56), HI only (aOR = 1.60, 95% CI 1.21–2.12), and DSI (aOR = 1.65, 95% CI 1.38–1.96) were associated with elevated risks. Mediation analysis revealed that depressive symptoms partially explained these associations, accounting for 18.28% of the VI–arthritis association, 12.70% for HI, and 15.69% for DSI. The findings were consistent across secondary analyses.

**Conclusion:**

The findings of this study highlight that sensory impairments are significantly associated with an increased risk of arthritis among older Chinese adults, with depressive symptoms serving as a partial mediator. These results underscore the importance of integrating mental health management into the care strategies for individuals with sensory disabilities. Future research should explore intervention strategies aimed at addressing both sensory impairments and mental health to further reduce the incidence of arthritis in this vulnerable demographic.

**Supplementary Information:**

The online version contains supplementary material available at 10.1186/s12889-025-25495-x.

## Introduction

With the intensification of China’s aging population, health problems among the elderly are becoming increasingly prominent, particularly the prevalence of vision and hearing impairments, which pose significant health challenges for this demographic [1]. Visual impairment (VI) typically refers to a reduction in visual capability, while hearing impairment (HI) denotes a decline in auditory ability. Dual sensory impairment (DSI) describes a condition in which both visual and hearing impairments are presentsimultaneously [2, 3].

As individuals age, the prevalence of DSI has risen markedly. Studies indicate that approximately 40% of elderly individuals over the age of 70 experience at least one sensory impairment, and the coexistence of multiple sensory impairment is common [4]. Furthermore, DSI are linked to an elevated risk of mortality; research has found that individuals with dual sensory impairments have a 44% higher risk of death compared to those without sensory impairments [5]. This underscores that dual sensory impairment is not only a significant factor affecting the quality of life for the elderly but is also closely associated with their survival rates.

Arthritis is primarily categorized into two subtypes: osteoarthritis (OA) and rheumatoid arthritis (RA) [6]. Among these, OA is the most prevalent joint disease in older adults and exhibits a significant positive correlation with age. As individuals age, the risk of developing osteoarthritis increases substantially, posing serious health threats to elderly populations [7, 8].In contrast, rheumatoid arthritis (RA) is an autoimmune disorder characterized by chronic erosive arthritis, predominantly affecting middle-aged and elderly women. This condition not only severely compromises joint functionality but also leads to joint deformities and mobility restrictions, consequently elevating overall disease burden and mortality risk in affected individuals [9, 10].

In recent years, research has increasingly focused on the relationship between sensory impairments and arthritis. A study based on the Irish Longitudinal Study on Ageing (TILDA) found that various eye diseases, including cataracts and glaucoma, significantly increase the likelihood of developing arthritis within two years [11]. Another cross-sectional study based on the National Health and Nutrition Examination Survey (NHANES) revealed that adults with arthritis reported significantly higher instances of vision, hearing, and balance impairments compared to participants without arthritis, particularly concerning visual impairments [12].

Dual sensory impairment not only undermines quality of life but is also closely linked to adverse psychosocial factors, including cognitive decline and heightened depressive symptoms, which may increase arthritis risk through psychological and inflammatory pathways [3, 13–15]. Prior studies consistently report strong associations between sensory impairment and depressive symptoms. Emerging longitudinal evidence suggests a directional, possibly bidirectional, link between depression and arthritis [16, 17]. Biologically, depression may activate the hypothalamic–pituitary–adrenal (HPA) axis and upregulate pro-inflammatory cytokines (e.g., IL-6, TNF-α), fostering low-grade systemic inflammation that promotes synovitis, pain sensitization, and cartilage degradation [18]. Behaviorally and socially, sensory loss can restrict social engagement, reduce physical activity, and worsen sleep, thereby aggravating depressive symptoms and compromising joint health [19–21]. Taken together, depressive symptoms are a plausible mediator between sensory impairment and arthritis; however, few studies have rigorously tested this hypothesis within a longitudinal, population-based framework.

Against this background, we used nationally representative data from the China Health and Retirement Longitudinal Study (CHARLS, 2011–2015) to examine the association of visual and hearing impairment with the risk of new-onset arthritis and to evaluate the mediating role of depressive symptoms.By embedding sensory function, mental health, and incident arthritis within a unified longitudinal framework, this study quantitatively assesses the proposed pathway “sensory impairment → depression → arthritis.” This approach extends prior research, which has predominantly been cross-sectional or focused on specific diseases, and provides population-level support for integrated strategies that combine sensory management with depression screening.

## Materials and methods

### Study population

**Fig. 1:**
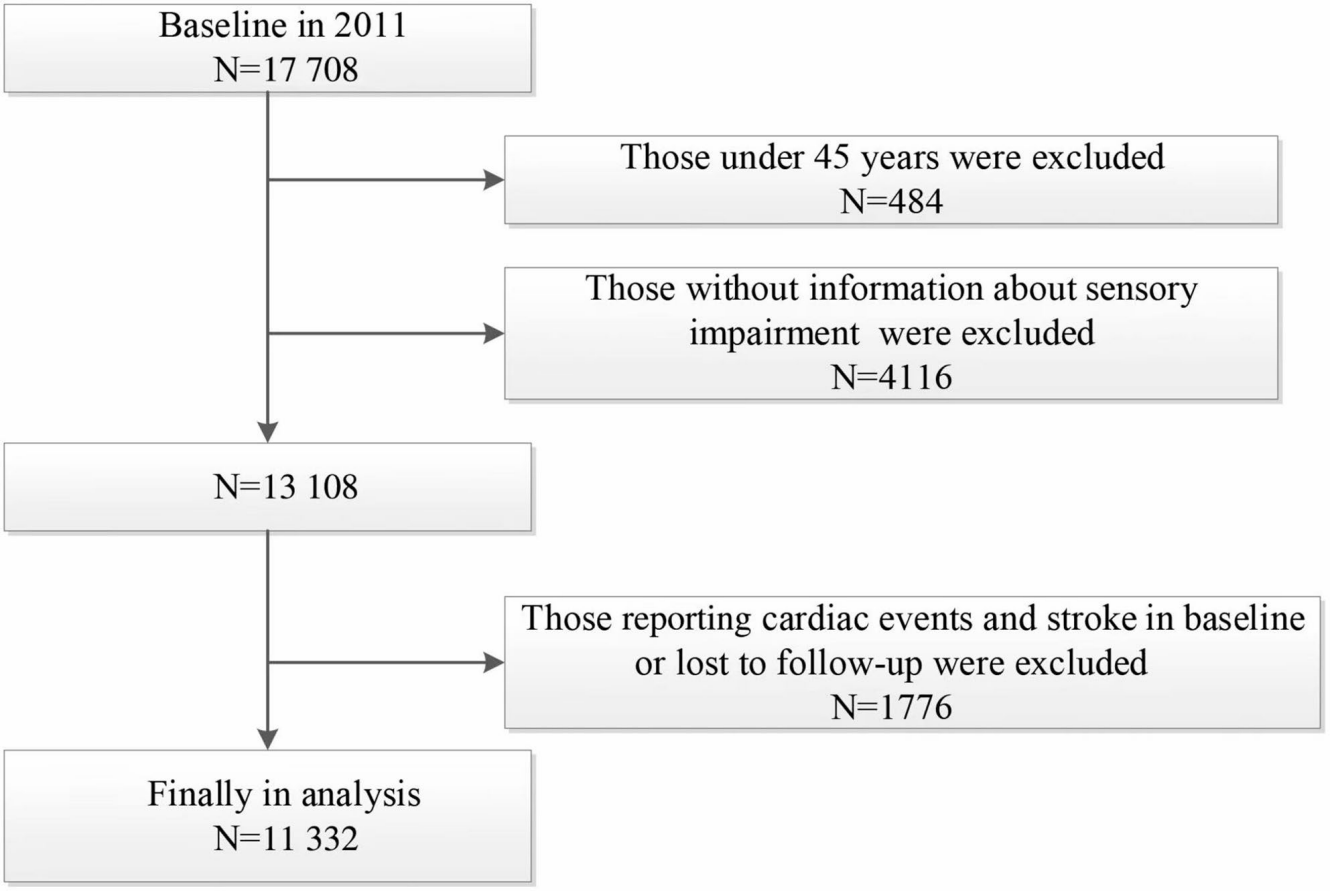
Flow chart of sample selection and the exclusion criteria. According to the figure, the number of observations was 8 415

The study population was derived from CHARLS, a nationally representative longitudinal survey involving Chinese adults aged 45 years and older. The baseline survey (2011) enrolled 17,708 participants, with biennial follow-ups conducted thereafter [22, 23]. Detailed CHARLS methodology has been previously documented [24–26]. Our 4-year longitudinal analysis (2011–2015) utilized 2011 data as baseline. After excluding participants with baseline arthritis diagnoses or incomplete arthritis information during follow-up, we prospectively monitored eligible subjects through 2015. From 17,708 initially screened participants, we excluded: 648 aged < 45 years, 1,378 with missing sensory impairment data, 208 with incomplete depression status, 34 from wave 1, 2,604 with incomplete wave 3 arthritis data, and 4,421 with confirmed baseline arthritis. The final analytical cohort consisted of 8,415 participants (Fig. 1), which included 6,861 individuals free of arthritis and 1,554 cases of new-onset arthritis.

CHARLS received ethical approval from Peking University’s Institutional Review Committee (IRB00001052-11015). All participants provided written informed consent prior to enrollment. This study adheres to STROBE reporting guidelines.

### Assessment of sensory impairment

HI and VI statuses were ascertained through standardized self-report questionnaires [27]. VI assessment incorporated two items: (1) “How would you rate your distance vision (with glasses/corrective lenses if used), such as recognizing a friend across the street?” and (2) “How would you assess your near vision (with glasses/corrective lenses if used), such as reading newspapers?” Responses for each item were categorized as: excellent, very good, good, fair, or poor. Participants reporting “fair” or “poor” in either category were classified as having VI[27].HI evaluation utilized the question: “How would you describe your hearing? (excellent/very good/good/fair/poor) Please specify hearing aid usage frequency if applicable.” Respondents indicating “fair” or “poor” hearing were categorized as HI cases [27]. Concurrent presence of both impairments defined DSI [27, 28].

### Assessment of new-onset arthritis

The diagnosis of new-onset arthritis was based on self-reported data; when the interviewer asked, “Have you been diagnosed with arthritis by a doctor?” and the respondents answered “Yes,” they were classified as arthritis patients. Subjects who had arthritis in 2011 were excluded, and if the patient was diagnosed with arthritis after that until the follow-up period in 2015, he or she was included in the study under our definition of a patient with new-onset arthritis [29].

### Assessment of depressive symptoms

The 10-item Center for Epidemiologic Studies Depression Scale (CES-D-10) assesses depressive symptoms experienced in the preceding week. This instrument contains two domains: eight negatively phrased items and two positively worded items. Respondents rated symptom frequency using a 4-point scale: 0 (less than one day), 1 (1–2 days), 2 (3–4 days), and 3 (5–7 days). Positive items were reverse-scored. Total scores (range: 0–30) were derived by summing all items, with higher scores indicating greater symptom severity. Depression status was dichotomized using the established cutoff ≥ 10 points [30].

### Assessment of covariates

Informed by published research and clinical judgment [27, 28].we adjusted for potential confounders through comprehensive covariate selection. Demographic covariates included: age (continuous), sex (male/female), residence (urban/rural), education (Illiteracy/Primary school/Middle school/High school or above), and marital status (Married or live together/other). Lifestyle factors comprised: smoking (never/former/current), drinking (never/former/current), sleep duration (hours), and body mass index (BMI). Chronic conditions included physician-diagnosed or clinically confirmed cases of hypertension, diabetes, chronic kidney disease, cardiovascular disease, and digestive diseases [31]. Anthropometric measurements were obtained following standard protocols: weight (kg) and height (cm) measured without shoes/heavy clothing. BMI was computed as weight (kg) divided by height squared (m²). Hypertension was defined as either self-reported physician diagnosis or measured blood pressure ≥ 140/90 mmHg. Diabetes required either self-reported diagnosis, fasting glucose ≥ 7 mmol/L, random glucose ≥ 11.1 mmol/L, or HbA1c ≥ 6.5% [31, 32].

###  Statistical analysis

In the dataset, we describe the baseline characteristics of non-normally distributed data: categorical variables are presented as totals and percentages, while continuous variables are expressed as medians and interquartile ranges (IQR). Intergroup comparisons are conducted using t-tests, Mann-Whitney U tests, or chi-square tests, depending on the type of data distribution.

We conducted a logistic regression analysis to investigate the prospective association between baseline sensory impairment and arthritis, expressed as odds ratios (OR) with 95% confidence intervals (CI). To mitigate the influence of confounding factors, we employed multivariable adjusted models. Specifically, Model 1 is a simple regression model without adjustment for confounding factors. Model 2 adjusts for sex, age, marital status, residence, educational level.; Model 3 further adjusts for drinking status, smoking status, sleep duration, and BMI based on Model 2. Model 4 further adjusts for chronic disease history, including hypertension, diabetes, chronic kidney disease, heart disease, and digestive diseases, based on Model 3. Model 5 further adjusts for depressive symptoms as measured by the CES-D-10 score based on Model 4 [31, 32].

In addition, we conducted a subgroup analysis to assess the potential interactions among various variables. Specifically, we examined the interactions based on the following factors: sex, age, marital status, residence, BMI, sleep duration, smoking status, drinking status, depressive symptoms, and chronic medical history (hypertension, diabetes, chronic kidney disease, heart disease, and digestive diseases) [33].The mediation model was constructed using the R package mediation, employing a bootstrap approach with 1,000 simulations [34]. In this analysis, depressive symptoms, measured at baseline (2011) using the CES-D-10 scale, were treated as the mediator, while incident arthritis between 2011 and 2015 was defined as the outcome. The model estimated the indirect effect (through depressive symptoms), direct effect (remaining after accounting for the mediator), and total effect of sensory impairments on arthritis risk. The proportion of mediation was calculated as the ratio of the indirect effect to the total effect, adjusting for the same covariates as in Model 4. This study utilized the R 4.4.2 statistical analysis software, considering a significance level of *P* < 0.05.

## Results

### Participants characteristics

Our study included 8,415 participants (4,296 males [51.05%] and 4,119 females [48.95%]). Participants were stratified into four groups based on sensory status: (1) No sensory impairment (*n* = 1,610, 19.13%), (2) Vision impairment only (*n* = 2,472, 29.37%), (3) Hearing impairment only (*n* = 467, 5.55%), and (4) Dual sensory impairment (*n* = 3,866, 45.95%).

Comparative analysis revealed that, in contrast to the no sensory impairment group, significant differences were particularly evident in age, sleep duration, BMI, and depression symptoms among the groups with vision impairment only, hearing impairment only, and dual sensory impairment (*p* < 0.001). The vision impairment only group, the hearing impairment only group and the dual sensory impairment group were significantly older and exhibited more depressive symptoms compared to the non-sensory impairment group. Additionally, the dual sensory impairment group experienced shorter sleep duration and had a lower BMI.Additionally, significant differences were observed in sex, marital status, education level, residence, drinking and smoking status, and chronic disease conditions(hypertension, chronic kidney disease, heart disease, and digestive diseases) (*p* < 0.05). For further details, please refer to Table 1.

**Table 1:** Presents the baseline characteristics of the study population categorized by sensory impairment in 2011

	Total (*n* = 8415)	No sensory impairment (*n* = 1610)	Vision impairment only (*n* = 2472)	Hearing impairment only (*n* = 467)	Dual sensory impairment(*n* = 3866)	*p*
Age, median (IQR), years	57 (51, 64)	55 (48, 62)	56 (50, 62)	59 (50, 68)	59 (53, 66)	< 0.001
Sleeptime, median (IQR), hours	7 (5, 8)	7 (6, 8)	7 (6, 8)	7 (6, 8)	6 (5, 8)	< 0.001
BMI (IQR), kg/m2	23.11 (20.89, 25.67)	23.32 (21.11, 25.89)	23.12 (21.07, 25.74)	23.38 (20.9, 26.49)	22.98 (20.68, 25.42)	< 0.001
CESD-10 scores, median (IQR)	6 (3, 11)	4 (2, 7)	6 (3, 10)	5 (3, 9)	8 (4, 12)	< 0.001
Sex, *n* (%)						< 0.001
Female	4119 (48.95)	703 (43.66)	1248 (50.49)	193 (41.33)	1975 (51.09)	
Male	4296 (51.05)	907 (56.34)	1224 (49.51)	274 (58.67)	1891 (48.91)	
Marriage status, *n* (%)						0.003
Others	938 (11.15)	145 (9.01)	260 (10.52)	57 (12.21)	476 (12.31)	
Married or live together	7477 (88.85)	1465 (90.99)	2212 (89.48)	410 (87.79)	3390 (87.69)	
Education level, *n* (%)						< 0.001
Illiteracy	3580 (42.58)	536 (33.31)	1008 (40.79)	194 (41.54)	1842 (47.71)	
Primary school	1808 (21.5)	319 (19.83)	527 (21.33)	84 (17.99)	878 (22.74)	
Middle school	1921 (22.85)	445 (27.66)	577 (23.35)	114 (24.41)	785 (20.33)	
High school or above	1099 (13.07)	309 (19.2)	359 (14.53)	75 (16.06)	356 (9.22)	
Residence, *n* (%)						< 0.001
Urban	3267 (38.82)	729 (45.28)	1032 (41.75)	184 (39.4)	1322 (34.2)	
Rural	5148 (61.18)	881 (54.72)	1440 (58.25)	283 (60.6)	2544 (65.8)	
Drinking, *n* (%)						< 0.001
Ex-drinking	653 (7.76)	111 (6.89)	155 (6.27)	48 (10.28)	339 (8.77)	
Current drinking	2931 (34.84)	614 (38.14)	864 (34.97)	169 (36.19)	1284 (33.23)	
Never drinking	4828 (57.39)	885 (54.97)	1452 (58.76)	250 (53.53)	2241 (58)	
Smoking, *n* (%)						0.002
Ex-smoking	725 (8.62)	115 (7.14)	190 (7.69)	50 (10.71)	370 (9.57)	
Current smoking	2780 (33.04)	572 (35.53)	828 (33.51)	160 (34.26)	1220 (31.56)	
Never smoking	4909 (58.34)	923 (57.33)	1453 (58.8)	257 (55.03)	2276 (58.87)	
Hypertension, *n* (%)						< 0.001
No	4690 (55.85)	984 (61.16)	1411 (57.13)	248 (53.33)	2047 (53.13)	
Yes	3707 (44.15)	625 (38.84)	1059 (42.87)	217 (46.67)	1806 (46.87)	
Diabetes, *n* (%)						0.724
No	7376 (88.17)	1423 (88.94)	2160 (87.84)	409 (87.58)	3384 (88.12)	
Yes	990 (11.83)	177 (11.06)	299 (12.16)	58 (12.42)	456 (11.88)	
Heart disease, *n* (%)						< 0.001
No	7612 (90.84)	1490 (92.78)	2282 (92.61)	417 (90.26)	3423 (88.96)	
Yes	768 (9.16)	116 (7.22)	182 (7.39)	45 (9.74)	425 (11.04)	
Kidney disease, *n* (%)						0.021
No	8032 (96)	1552 (96.88)	2371 (96.3)	450 (96.98)	3659 (95.31)	
Yes	335 (4)	50 (3.12)	91 (3.7)	14 (3.02)	180 (4.69)	
Digestive diseases, *n* (%)						< 0.001
No	6995 (83.32)	1421 (88.37)	2061 (83.64)	395 (84.76)	3118 (80.84)	
Yes	1400 (16.68)	187 (11.63)	403 (16.36)	71 (15.24)	739 (19.16)	
Depressive symptoms, *n* (%)						< 0.001
No	5869 (69.74)	1345 (83.54)	1806 (73.06)	359 (76.87)	2359 (61.02)	
Yes	2546 (30.26)	265 (16.46)	666 (26.94)	108 (23.13)	1507 (38.98)	

### Longitudinal association between sensory impairment and new risk of arthritis

During the 4-year follow-up, 1,554 participants developed arthritis. In the fully adjusted model, VI was associated with increased arthritis risk compared to unimpaired counterparts (OR = 1.32, 95%CI = 1.14–1.52). Similarly, HI demonstrated elevated risk relative to non-HI participants (OR = 1.39, 95%CI = 1.23 ~ 1.56). Participants with DSI had significantly higher arthritis risk (OR = 1.34, 95%CI = 1.19 ~ 1.51). Notably, DSI showed 65% greater risk compared to those no sensory impairment (OR = 1.65, 95%CI = 1.38–1.96).**(**Table 2.**)**For transparency, the complete coefficients for all covariates in the fully adjusted model are provided in Supplementary (Table S1).

**Table 2:** Association between sensory impairment and new-onset arthritis

Variables	Model1		Model2		Model3		Model4		Model5	
	OR (95%CI)	*P*	OR (95%CI)	*P*	OR (95%CI)	*P*	OR (95%CI)	*P*	OR (95%CI)	*P*
Vision impairment										
No	1.00 (Reference)		1.00 (Reference)		1.00 (Reference)		1.00 (Reference)		1.00 (Reference)	
Yes	1.58 (1.38 ~ 1.82)	< 0.001	1.47 (1.28 ~ 1.70)	< 0.001	1.43 (1.24 ~ 1.65)	< 0.001	1.40 (1.21 ~ 1.62)	< 0.001	1.32 (1.14 ~ 1.52)	< 0.001
Hearing impairment										
No	1.00 (Reference)		1.00 (Reference)		1.00 (Reference)		1.00 (Reference)		1.00 (Reference)	
Yes	1.59 (1.42 ~ 1.77)	< 0.001	1.50 (1.34 ~ 1.68)	< 0.001	1.47 (1.31 ~ 1.66)	< 0.001	1.46 (1.29 ~ 1.64)	< 0.001	1.39 (1.23 ~ 1.56)	< 0.001
Dual sensory impairment										
No	1.00 (Reference)		1.00 (Reference)		1.00 (Reference)		1.00 (Reference)		1.00 (Reference)	
Yes	1.57 (1.40 ~ 1.75)	< 0.001	1.47 (1.32 ~ 1.65)	< 0.001	1.44 (1.29 ~ 1.62)	< 0.001	1.42 (1.26 ~ 1.59)	< 0.001	1.34 (1.19 ~ 1.51)	< 0.001
Sensory impairment										
No sensory impairment	1.00 (Reference)		1.00 (Reference)		1.00 (Reference)		1.00 (Reference)		1.00 (Reference)	
Vision impairment only	1.45 (1.21 ~ 1.74)	< 0.001	1.39 (1.15 ~ 1.67)	< 0.001	1.36 (1.13 ~ 1.64)	0.001	1.34 (1.11 ~ 1.62)	0.002	1.29 (1.07 ~ 1.56)	0.008
Hearing impairment only	1.68 (1.27 ~ 2.21)	< 0.001	1.63 (1.24 ~ 2.16)	< 0.001	1.64 (1.24 ~ 2.16)	< 0.001	1.63 (1.23 ~ 2.16)	< 0.001	1.60 (1.21 ~ 2.12)	0.001
Dual sensory impairment	2.05 (1.74 ~ 2.43)	< 0.001	1.88 (1.59 ~ 2.23)	< 0.001	1.82 (1.53 ~ 2.16)	< 0.001	1.78 (1.50 ~ 2.12)	< 0.001	1.65 (1.38 ~ 1.96)	< 0.001

Model 1: No adjustments made

Model 2: Adjusted for age, sex, residence, marriage status and educational level

Model 3: Adjusted for age, sex, residence, educational level, marriage status, body mass index, sleep duration, smoking status, and drinking status

Model 4: Adjusted for age, sex, residence, educational level, marriage status, body mass index, sleep duration, smoking status, drinking status, and chronic medical history (diabetes, hypertension, chronic kidney disease, heart disease, and digestive diseases)

Model 5: Adjusted for age, sex, place of residence, educational level, body mass index, sleep duration, smoking status, drinking status, chronic medical history (diabetes, hypertension, chronic kidney disease, heart disease, and digestive diseases), and CES-D-10 scores

### Subgroup analysis

To investigate the relationship between visual impairment (VI), hearing impairment (HI), dual sensory impairment (DSI), and the new-onset arthritis, we categorized participants into distinct subgroups based on sociodemographic variables and medical histories, subsequently conducting interaction analysis.

**Fig. 2:**
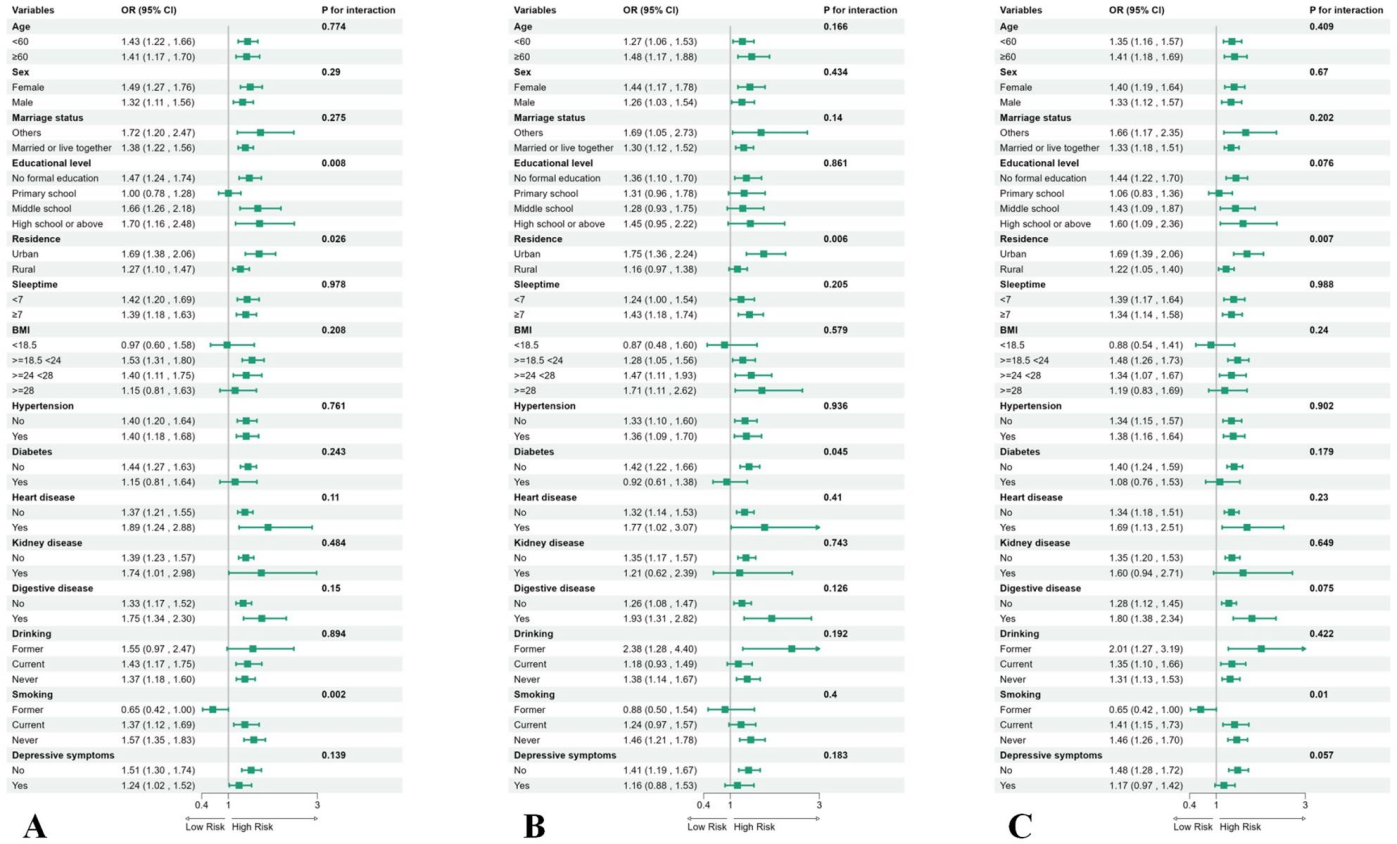
Forest plot of stratified analysis of the correlation between VI (**A**), HI (**B**), DSI (**C**) and the risk of new-onset arthritis

In examining the association between visual impairment (VI) and arthritis, we found that residential area and diabetes status significantly interacted with the outcome of new arthritis. The odds ratio (OR) for urban residents was 1.75 (95% CI: 1.36–2.24), which was greater than that for rural residents (OR: 1.16, 95% CI: 0.97–1.38). Additionally, the OR for non-diabetic individuals was 1.42 (95% CI: 1.22–1.66), surpassing that for individuals with diabetes (OR: 0.92, 95% CI: 0.61–1.38) **(**Fig. 2.A).

In the relationship between HI and arthritis, three variables—education level, living area, and smoking status—showed significant interactions. The odds ratios (ORs) across educational attainment were: 1.47 (95%CI:1.24–1.74) for < high school, 1.00 (95%CI:0.78–1.28) for high school graduates, and 1.66 (95%CI:1.26–2.18) for some college education. For those with ≥ high school education, the OR was 1.70 (95%CI:1.16–2.48). Urban residents had higher risk (OR = 1.69, 95%CI:1.38–2.06) than rural residents (OR = 1.27, 95%CI:1.10–1.47). Smoking subgroups showed ORs of 0.65 (95%CI:0.42–1.00.42.00) for former smokers, 1.41 (95%CI:1.15–1.73) for current smokers, and 1.46 (95%CI:1.26–1.70) for never smokers.(Fig. 2.B).

The relationship between DSI and arthritis showed significant interactions by residence and smoking status. Urban residents had OR = 1.69 (95%CI:1.39–2.06) versus rural residents OR = 1.22 (95%CI:1.05–1.40). Smoking subgroups demonstrated ORs of 0.65 (95%CI:0.42–1.00.42.00) for former smokers, 1.41 (95%CI:1.15–1.73) for current smokers, and 1.46 (95%CI:1.26–1.70) for never smokers.(Fig. 2.C).

In the multivariate model, adjustments were made for confounders including age, sex, place of residence, educational level, body mass index, sleep duration, smoking status, drinking status, chronic medical history (diabetes, hypertension, chronic kidney disease, heart disease, and digestive diseases), and depressive symptoms.

### Mediation analysis

To further evaluate the mediating role of depressive status in the relationship between sensory impairments and the risk of arthritis, this study employs the CESD-10 as a mediating variable. We controlled for factors such as age, sex, residence, education level, body mass index, sleep duration, smoking status, drinking status, and chronic medical history (diabetes, hypertension, chronic kidney disease, heart disease, and digestive system diseases). The results of the mediation analysis indicate that depressive symptoms partially mediate the relationship between sensory impairments and the risk of arthritis. Specifically, the indirect effects of visual impairment, hearing impairment, and dual sensory impairment on the risk of arthritis through depressive symptoms account for 18.28%, 12.70%, and 15.69% of the total effect, respectively (Table 3).

**Table 3:** The association between CES-D-10 scores and the mediation of VI, HI and DSI with new-onset arthritis

		Estimate	95% CI Lower	95% CI Upper	*P*-value
VI	ACME (average)	0.00832	0.00572	0.01	< 0.001 ***
	ADE (average)	0.03768	0.01867	0.06	< 0.001 ***
	Prop. Mediated (average)	0.18275	0.10886	0.32	< 0.001 ***
HI	ACME (average)	0.00701	0.00464	0.01	< 0.001 ***
	ADE (average)	0.0478	0.03058	0.07	< 0.001 ***
	Prop. Mediated (average)	0.127	0.0778	0.21	< 0.001 ***
DSI	ACME (average)	0.00807	0.00536	0.01	< 0.001 ***
	ADE (average)	0.04334	0.02662	0.06	< 0.001 ***
	Prop. Mediated (average)	0.15687	0.09458	0.26	< 0.001 ***

## Discussion

This national study identified significant associations between sensory impairments and the risk of arthritis among Chinese middle-aged and older adults. Individuals with sensory disorders demonstrated substantially higher arthritis incidence rates compared to unimpaired peers. These associations persisted after comprehensive adjustment for demographic, lifestyle, and health-related confounders.VI, HI, and DSI each exhibited independent positive associations with arthritis risk. Mediation analyses revealed depressive symptoms mediated 18.28% (VI), 12.70% (HI), and 15.69% (DSI) of these associations.

Our CHARLS-based findings corroborate previous observations from an established European cohort showing visual impairment’s association with elevated arthritis risk[11]. Previous studies have rarely investigated how visual impairment, hearing impairment, and dual sensory impairment impact the development of new-onset arthritis. Moreover, the potential mediating role of depression in the link between sensory impairment and new-onset arthritis has not been adequately explored. After fully controlling for confounding factors such as demographic characteristics, lifestyle, and chronic disease history, this study found that depression plays a partial mediating role in the relationship between “visual and hearing impairment and the risk of new-onset arthritis.” This finding is supported at both theoretical and empirical levels.

From a theoretical perspective, depression may influence joint health through psychoneuroimmunological pathways. Persistent depressive states can lead to sustained activation of the hypothalamic–pituitary–adrenal (HPA) axis and increased secretion of pro-inflammatory cytokines such as IL-6, TNF-α, and CRP, resulting in a state of chronic low-grade inflammation that promotes synovitis, pain sensitization, and cartilage degradation [35, 36]. Previous studies have shown that IL-6 is not only involved in the pathogenesis of rheumatoid arthritis but is also closely associated with the development of depressive symptoms. These two conditions may interact through shared inflammatory signaling networks, forming a biological cascade of ‘emotion–inflammation–joint pathology.‘ [35, 37]. Systematic reviews and meta-analyses have further demonstrated that peripheral inflammatory markers are significantly elevated in patients with depression, introducing the concept of ‘inflamed depression’ and providing molecular-level evidence for the inflammatory effects mediated by depression [38, 39].On the psychosocial and behavioral level, visual or hearing impairments often lead to communication difficulties and reduced social interaction, which can increase feelings of loneliness and social isolation, thereby precipitating or exacerbating depression. Longitudinal studies have confirmed robust associations between sensory impairments and depressive symptoms, with functional limitations and insufficient social support serving as potential mediators [14, 15]. Individuals with dual sensory impairments face a significantly higher risk of depression compared to those with a single sensory deficit [14, 15, 40].At the population level, longitudinal studies among middle-aged and older Chinese adults have revealed a close, and possibly bidirectional, relationship between depression and arthritis: persistent depressive trajectories predict the subsequent onset of arthritis, while chronic pain and functional limitations resulting from arthritis further exacerbate depressive symptoms, creating a mutually reinforcing cycle [16, 17, 41]. Meta-analytic evidence also indicates that individuals with depression have an increased risk of developing rheumatoid arthritis, and that the prevalence of depression among patients with rheumatoid arthritis is substantially higher than in the general population [38, 41].

The mediation analysis conducted in this study further substantiates the aforementioned hypothesis. Utilizing the 2011 baseline CES-D-10 depression score as the mediator and new-onset arthritis from 2011 to 2015 as the outcome, we employed a bootstrap resampling procedure (1,000 iterations) to estimate the indirect effects and the proportion mediated. The results indicated significant indirect effects of depression in the associations between visual, hearing, and dual sensory impairments and new-onset arthritis, with mediation proportions of 18.3%, 12.7%, and 15.7%, respectively, all suggesting partial mediation. After adjusting for depressive symptoms, the direct effects of sensory impairment on new-onset arthritis remained significant, indicating that, in addition to the psychological pathway, parallel mechanisms—such as inflammatory responses, mechanical loading, and health-related behaviors—may also contribute. These findings align with previous studies and further reinforce the role of depression as a critical psychological and physiological mediator linking sensory impairment to arthritis risk.

The underlying mechanisms linking VI and HI to the onset of arthritis can be elucidated through several key aspects: Physical Activity Limitations: Patients with Visual Impairment (VI) and Hearing Impairment (HI) face numerous mobility challenges in their daily lives, which significantly restrict their range and intensity of physical activity, thereby affecting their overall level of physical activity [42]. Prolonged inactivity can lead to decreased joint flexibility and weakened muscle strength around the joints [43], resulting in abnormal loads and excessive mechanical stress on the joints, which is closely related to the development of osteoarthritis (OA) [44, 45].Cognitive impairment: Both VI and HI are closely associated with cognitive impairment. Research has indicated that individuals with DSI exhibit a significantly elevated risk of cognitive decline [46]. This decline in cognitive function not only disrupts various aspects of daily life but also correlates positively with the risk of developing arthritis [47]. Cognitive impairment may result in reduced attention to joint health, an inability to promptly recognize subtle joint discomfort, and challenges in implementing effective preventive and protective measures, thereby increasing the likelihood of joint disease [48, 49].Impact of related diseases: Certain diseases closely associated with DSI, such as Cardiovascular Disease (CVD), significantly influence the relationship between VI, HI, and the onset of new arthritis. Research indicates that patients with DSI exhibit a markedly higher risk of developing cardiovascular disease compared to those with either single sensory disorders or no sensory disorders [50]. There exists a notable intersection between CVD and arthritis in their pathogenesis. These shared pathophysiological pathways may interact to enhance the occurrence and progression of arthritis, rendering DSI patients more susceptible to its onset, thereby significantly increasing the risk of new arthritis [51, 52].

VI and HI identified in this study constitute modifiable risk factors for incident arthritis, underscoring the importance of early intervention strategies. For individuals with VI, implementing regular ophthalmologic evaluations enables timely correction of refractive errors through prescription lenses, while condition-specific management should be provided for cataracts and other ocular pathologies [53, 54]. Concurrently, enhancing joint protection education programs can improve patients’ safety awareness and self-management capacity in daily activities.In HI populations, routine audiological assessments with appropriate hearing aid provision should be integrated with mental health monitoring and social support enhancement [55]. Encouraging active social participation has demonstrated efficacy in alleviating psychological distress [56, 57], which may be further reinforced through targeted arthritis education utilizing adapted materials such as visual guides and sign language resources.

This study represents the first attempt to integrate sensory impairment, depressive symptoms, and new-onset arthritis into a unified longitudinal framework within a nationally representative Chinese cohort, quantifying the proportion of the effect mediated by depression. In contrast to previous evidence that has predominantly been cross-sectional or disease-specific, our findings offer population-based insights into the potential causal pathways linking sensory and joint health. From both public health and clinical perspectives, the results underscore the necessity of incorporating depression screening and intervention into the management of vision and hearing health among older adults. The integration of routine visual and auditory assessments, alongside CES-D-10 depression screening, into standard medical examinations may contribute to a reduction in the risk of developing arthritis and provide a practical approach for early prevention and comprehensive intervention.

This study is based on the CHARLS study, a nationally representative longitudinal cohort study. The research has several advantages.The sample size used in this study is sufficiently large, ensuring robust statistical power, and CHARLS also has a nationally representative sample. By introducing depressive symptoms as a mediator, this study attempts for the first time to establish the association between depressive symptoms, sensory impairments, and the risk of arthritis in the Chinese population.However, stating certain inherent limitations in this study is necessary. Firstly, the judgments of visual impairment (VI) and hearing impairment (HI) are primarily based on participants’ self-reports, which may introduce information bias. Additionally, our study did not track the dynamic changes in sensory impairment status during the follow-up period, which could affect the analysis. Secondly, the CES-D10 questionnaire relies on self-reports from the sampled population, potentially introducing risks of recall bias and inaccuracies in responses. While the CES-D10 is a widely used instrument for assessing clinically significant depressive symptoms, it serves solely as a screening tool and is not intended for diagnosing depression. Furthermore, given that depression frequently coexists with mental comorbidities such as anxiety disorders, and the CHARLS database does not systematically collect diagnostic information regarding these comorbidities, we are unable to account for their potential confounding effects. Third, the CHARLS subjects consist of individuals aged 45 and older; however, no data was collected regarding their prior health conditions, which may influence health statuses that could subsequently affect the risk of developing arthritis.Fourthly, although we employed nationally representative data, caution is warranted when extrapolating findings to non-Chinese populations due to genetic and environmental heterogeneity. Finally, despite extensive covariate adjustments, residual confounding from unmeasured factors (e.g., dietary patterns, lifestyle modifications) persists.

## Conclusion

This study demonstrates a positive correlation between sensory disorders and the risk of arthritis in Chinese populations. Additionally, symptoms of depression serve as a significant mediating factor in this relationship. These findings indicate that arthritis prevention strategies should prioritize both the monitoring of sensory disorders and the management of depressive symptoms.

## Supplementary Information

Supplementary Material 1.

## Data Availability

The datasets supporting the conclusions of this article are available publicly, http://charls.pku.edu.cn/pages/data/111/en.html.

## Abbreviations

CHARLS: China Health and Retirement Longitudinal Study
OR: Odds ratio
CI: Confidence interval
VI: Visual impairment
HI: Hearing impairment
DSI: Dual sensory impairment
CES-D-10: Center for Epidemiologic Studies Depression Scale (10-item version)
BMI: Body mass index
CVD: Cardiovascular disease
RA: Rheumatoid arthritis
OA: Osteoarthritis
NHANES: National Health and Nutrition Examination Survey
TILDA: The Irish Longitudinal Study on Ageing
ACME: Average Causal Mediation Effect
ADE: Average Direct Effect
